# A Nurses' Charter of Liberty

**Published:** 1918-08-24

**Authors:** 


*To the Editor of* The Hospital.

### A NURSES' CHARTER OF LIBERTY.

*To the Editor of* The Hospital.

Sir,—May I offer a few remarks in reply to "Ierne." She (?) seems to think because there are so few replies to her (?) letters that nurses and sisters are either frightened to answer or are not interested in any way regarding their future or the future of those nurses who will follow them.

Does it not occur to her (?) that probably the nurses are quite happy in the lot they have chosen, and not so dissatisfied as she (?) would like one to think? I have spent twenty years in hospital, ten of which were spent as sister and five as matron. I would like to say I have never felt so overworked or underpaid. The work is often very hard indeed; personally, I have been on duty over twenty-four hours without a break—many times fifteen and sixteen hours, but I have always felt this to be a necessity for the time being when an extra rush of work has been on. Apart from those extra times, I do not think the work is any harder than other callings where a woman has a living to get. I would like, if possible, for the hours of duty to be shorter, but I certainly would not like to think my nurses had only eight hours work a day. One cannot but wonder what young women would be doing with eight hours a day on hand, usually with no near friends. I do not think they would be happy or even safe; besides, they would require a longer time for training.

Nurses seldom if ever make any friends in the town where their work lies; that, to my point of view, is one of the greatest drawbacks of hospital life—want of outside friends and companionship. I have known nurses who have spent the whole of their monthly day off in bed, for the want of a friend or companion to spend the day with. It would be nice if the general public would realise that in every town, great or small, women are employed night and day nursing their sick, and treat them as guests of the town. Nurses do not need clubs, etc. A nurse wants a change of companionship altogether when off duty. I think every nurse, besides the usual off-duty time of two hours daily, a long Sunday alternately, a day a month, and three weeks holiday a year, should be allowed one long evening a week from 5 to 11 P.M. This to allow her time to go to a concert or theatre at her own will, without having to ask for a late pass. It is degrading for a woman not to have one evening of her own to arrange for pleasure without permission.

As regards the payment of nurses. All things have to be considered. To begin, no special qualifications are needed to enter a training-school; refinement and a good ordinary education are all that is necessary. She has no entrance fee to pay. Her lectures are given free of charge. In fact, she is trained to a profession which will bring her a comfortable living for life. During her training she is provided with everything, even her uniform to work in. I take it that board, lodging, washing, uniform, baths, attendance, use of piano, etc., are worth at the very least £1 a week to any woman. Thus a probationer with £12 first year, £15 second year, £18 third year, will be seen to be earning £64 first year, £67 second year, and £70 third year. I doubt if there is any other profession or business where an unqualified person can earn more money. If there is, I certainly have not heard of it.

As regards the pay of trained nurses, I consider a start of £45 a year, rising to £55 a year, good pay, except where the nurse holds special certificates such as massage, maternity, dispensing. For such cases she should certainly be paid at a higher rate than the ordinary trained nurse. As it is usual for these special certificates to be obtained at the nurses' own expense, they should at the least have £10 a year extra. A trained nurse will thus be seen to be earning, with board, lodging, washing, uniform, baths, attendance, etc., valued at £52 per annum; salary £45 to £55 per annum, practically £100 a year. And with this she has no working clothes to find. I do not know any other calling in life where

**THE EDITOR'S LETTER-BOX—(*continued*).**

a woman can earn more, only in those professions where a large entrance fee has been paid to obtain their knowledge, and a large amount of the salary obtained has to be spent in keeping up appearances in dress. This ought to be taken into consideration. A nurse gets a free training—the cost to her is nil.

I would like a "living-out" system for trained nurses. A nurse is quite capable of coming and going to and from her work, the same as any other woman. She would benefit more by her earnings, as she could then take her money in value and not in necessities supplied. It is not that the nurse is so underpaid. It is liberty to spend her own that is wanted. All overtime work should be paid for, and when a nurse stays on duty after her usual hours, her time should be booked, and she should receive her extra money with her usual monthly pay.

I am sure any matron would be glad to alter many of the old-standing arrangements of hospital life (there are a few she would like altered for herself). If someone would only suggest a way to obtain more money for institutions, so that a matron's life need not be spent in trying to keep down expenses, and committees would recognise that it is not always policy to keep them down, and to work a place as cheaply as possible! It would be very nice to have an eight-hour day for matrons, but that would mean two matrons instead of one. Eight-hour days for sisters and nurses would be nice, but it would need a third of the staff extra. Can anyone suggest where the money is coming from to do this? A larger building would be required for accommodation, also extra maids. It is money that is wanted first, then discussion.

To finish. I do not think nursing such a hard lot. It is usually the chosen work of the nurse. And one is generally happy in such work. I sincerely hope a few more matrons will give their thoughts to the points I have raised and say if their lives have been too hard or underpaid for them. "Ierne" must not forget that all matrons have been nurses.—Yours,

August 18, 1918. MATRON.

